# Methods and Applications of In Silico Aptamer Design and Modeling

**DOI:** 10.3390/ijms21228420

**Published:** 2020-11-10

**Authors:** Andrey A. Buglak, Alexey V. Samokhvalov, Anatoly V. Zherdev, Boris B. Dzantiev

**Affiliations:** 1 A. N. Bach Institute of Biochemistry, Research Center of Biotechnology, Russian Academy of Sciences, Leninsky prospect 33, 119071 Moscow, Russia; 03alexeysamohvalov09@gmail.com (A.V.S.); zherdev@inbi.ras.ru (A.V.Z.); boris.dzantiev@mail.ru (B.B.D.); 2 Physical Faculty, St. Petersburg State University, 7/9 Universitetskaya naberezhnaya, 199034 St. Petersburg, Russia

**Keywords:** aptamers, in silico design, molecular modeling, docking, molecular dynamics

## Abstract

Aptamers are nucleic acid analogues of antibodies with high affinity to different targets, such as cells, viruses, proteins, inorganic materials, and coenzymes. Empirical approaches allow the design of in vitro aptamers that bind particularly to a target molecule with high affinity and selectivity. Theoretical methods allow significant expansion of the possibilities of aptamer design. In this study, we review theoretical and joint theoretical-experimental studies dedicated to aptamer design and modeling. We consider aptamers with different targets, such as proteins, antibiotics, organophosphates, nucleobases, amino acids, and drugs. During nucleic acid modeling and in silico design, a full set of in silico methods can be applied, such as docking, molecular dynamics (MD), and statistical analysis. The typical modeling workflow starts with structure prediction. Then, docking of target and aptamer is performed. Next, MD simulations are performed, which allows for an evaluation of the stability of aptamer/ligand complexes and determination of the binding energies with higher accuracy. Then, aptamer/ligand interactions are analyzed, and mutations of studied aptamers made. Subsequently, the whole procedure of molecular modeling can be reiterated. Thus, the interactions between aptamers and their ligands are complex and difficult to understand using only experimental approaches. Docking and MD are irreplaceable when aptamers are studied in silico.

## 1. Introduction

Aptamers are single-stranded nucleic acids (both DNA and RNA) with a high affinity toward target molecules. In the past few years, aptamers have been obtained for a wide range of targets, for example, cells, viruses, inorganic materials, metal ions, coenzymes, nucleobases, amino acids, antibiotics, pesticides, polypeptides, and hormones and other low-molecular-weight molecules [1,2,3]. Multiple studies have been performed that are dedicated to the development of biosensors functionalized with aptamers; the biosensors are based on various nanomaterials: quantum dots, metal nanoparticles (NPs), and single- and multiwalled carbon nanotubes [4,5,6].

**Figure 1:**
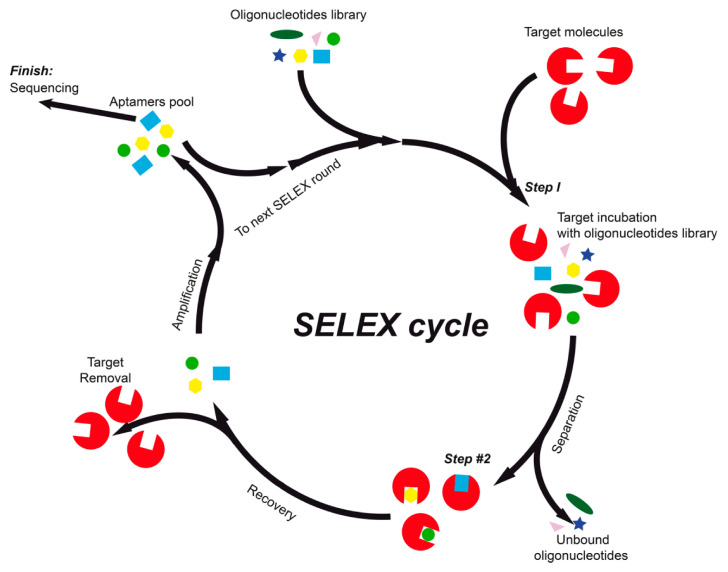
Schematic representation of SELEX aptamer selection and enrichment cycle.

The systematic evolution of ligands by exponential enrichment, or SELEX, is an experimental method for the determination of nucleic acid aptamers that specifically bind to a target molecule with high affinity and selectivity [7]. The construction of aptamers using SELEX technology includes several rounds of selection and enrichment processes (Figure 1), which are time and labor consuming and have a low cost-efficiency rate and output. SELEX may be combined with high-throughput sequencers, a process that is usually called HT-SELEX, or HTS [8,9].

Currently, there are several varieties of SELEX methodologies [10]. Two major steps exist in aptamer design selection and optimization. In the first step, several polynucleotides with probable binding affinity toward a target are screened by using the SELEX method and then selected. In the second step, aptamers with detected high affinity are shortened, modified, and stabilized. Both DNA and RNA aptamers can be designed. The usage of noncanonical nucleotides is possible [11].

An alternative strategy to SELEX that has been proposed in the past decade and a half is to apply computational methods of bioinformatics, namely, docking and molecular dynamics (MD), to design aptamers for various purposes. Of course, the in silico approach can be used along with SELEX and HTS to raise the efficacy of research. Some in silico tools are specialized for the analysis of HTS experimental data [12]. Rapid expansion and integration of next-generation sequencing (NGS) opens the possibility for conducting new high-throughput experiments and developing new screening strategies [13].

Hamada [14] highlighted several methods of oligonucleotide aptamer design: scalable clustering for RNA aptamers and motif-finding methods as well as aptamer optimization methods. An up-to-date review of aptamer design software is summarized in the paper by Emami et al. [15]. Additionally, new scoring functions are being developed, such as that in the study by Yan and Wang [16]. In silico aptamer design is usually performed with docking and MD. Sometimes, empirical research is carried out with only one technique, for example, MD. Sometimes, MD of oligonucleotide aptamers is accompanied by hybrid quantum mechanics/molecular mechanics (QM/MM) studies [17]. The quantitative structure–activity relationship (QSAR) method can be used along with other molecular modeling methods for aptamer design [18]. Thus, a full set of molecular modeling in silico tools can be exploited for aptamer design. Moreover, mathematical modeling of ligand/aptamer interaction kinetics is feasible [19,20].

A riboswitch is a noncoding RNA (ncRNA) that performs the function of genetic “switching” and regulates gene expression in response to a specific molecular target. Riboswitches are regulatory RNA components, which are usually located in the 5′-untranslated region of certain mRNAs and control gene expression during transcription or translation. These components consist of a sensor domain and a neighboring actuator domain. The sensor domain is an aptamer that specifically binds to a ligand, and the actuator domain includes an intrinsic terminator or a ribosomal binding site for transcriptional or translational regulation, respectively. A large part of the research dedicated to the design of oligonucleotide aptamers belongs to the development of RNA riboswitches [21,22,23,24,25].

**Figure 2:**
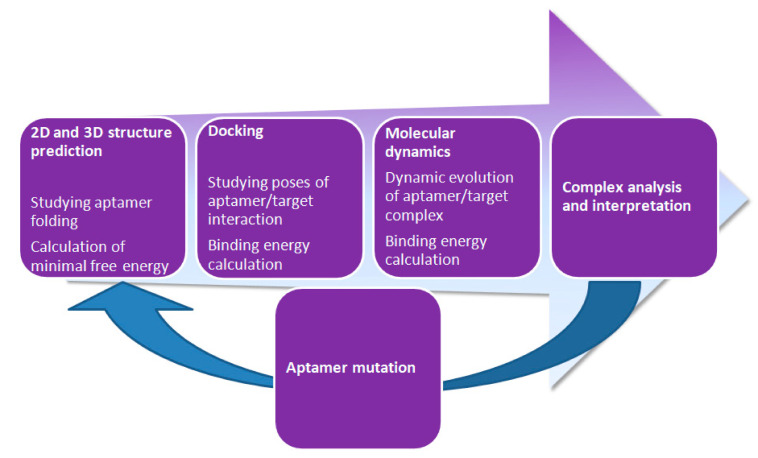
Typical workflow of aptamer in silico design and analysis.

In silico tools provide a wide range of methods to a researcher. It is possible to design aptamers using simple compounds as targets as well as complex biopolymers, such as proteins. The single disadvantage is that the current level of molecular modeling makes it impossible to use cells as targets in aptamer design, which is a plus of the in vitro SELEX method. Using molecular modeling methods, it is possible to find new aptamers with improved affinity to the target and to identify structural patterns responsible for aptamer/target interaction, which is helpful because point mutations may improve aptamer affinity. A typical modeling workflow of an aptamer/ligand in silico study is presented in Figure 2. The workflow starts with secondary structure prediction and then proceeds to tertiary structure optimization. Subsequently, rigid or flexible docking of the target and aptamer is performed, and then the complexes with the lowest binding energies are selected. The next important, but not mandatory, step is to perform molecular dynamic simulations, which allow for an evaluation of the stability of the aptamer/ligand complex and determination of the binding energies with higher accuracy than would have been possible during the previous docking step. Then, aptamer/ligand interactions are analyzed, which allows researchers to make mutations or even chemical modifications to the studied aptamers. After the analysis step, the whole procedure of the in silico aptamer study can be repeated.

In the next few sections, we summarize several studies dedicated to in silico aptamer design and modeling based on the type of aptamer targets used. Ligand molecules of aptamer design can be divided into several classes: peptides (especially surface glycoproteins, which allow the use of cells as targets in aptamer design), antibiotics, and organophosphates and other low-molecular-weight compounds. The aim and methodology of the study is different for various targets. For this reason, we grouped the studies based on the type of aptamer target used. The uniqueness of our review is due to the fact that we concentrated on concrete applications of in silico aptamer modeling and design, not on aptamer modeling methods nor modeling software.

## 2. Proteins as Targets of Aptamer Design

Proteins are probably the most popular targets of aptamer design and modeling. Short summaries of the computational methods and software used in these studies and descriptions of the main point of each study are presented in Table 1. Some of the proteins are autonomous analytes (e.g., thrombin), some of them are elements of polymolecular complexes, and some are cell surface proteins (e.g., hepatitis B surface antigen). The differences between protein structure and function influence the aptamer structure and design. In some studies, in silico design is dedicated to affinity modulation; in others, the aim is more therapeutic, and the main parameter is aptamer/ligand stability.

The aim of numerous in silico investigations was to improve aptamer-binding affinity using different methodologies in theoretical and joint theoretical-experimental studies. A second goal was to find structural patterns responsible for aptamer/protein binding. Some studies were dedicated to the approbation of new computational techniques for aptamer design. The most popular protein target of in silico aptamer design is thrombin. Inhibition of thrombin is important because it allows modulation of coagulation. The HIV1 proteins integrase and reverse transcriptase are also popular and prospective ligands because the development of such aptamers allows antiretroviral therapy to be performed and inhibition of virus DNA integration into the host genome. However, thrombin and HIV1 proteins were popular targets in the past. In the past few years, epithelial cell adhesion molecule (EpCAM)—a marker for carcinoma—became a prospective target, and many more in silico studies dedicated to this glycoprotein should be expected in the future.

We divided proteins as targets of aptamer design into four classes: (1) coagulation-related proteins, (2) infection-related proteins, (3) cancer-related proteins, and (4) other proteins.

### 2.1. Coagulation-Related Proteins

Thrombin (EC 3.4.21.5) is a 37-kDa serine protease that converts soluble fibrinogen into insoluble fibrin. The regulation of thrombin enzymatic activity offers the possibility of controlling blood coagulation. Thrombin-binding oligonucleotide aptamers have been used to inhibit thrombin activity. A ssDNA aptamer was designed toward thrombin protein using the entropic fragment-based approach (EFBA) [26]. EFBA was used to determine the probability distribution of the nucleobase sequences that most likely interact with the target protein. At the same time, sequences and corresponding tertiary structures were defined. EFBA included three steps: (1) determination of the probability distribution of a favored first nucleobase, (2) determination of the probability distribution of preferred neighboring nucleobases given the probability distribution of the favored nucleobase that was established at step 1, and (3) application of the entropic criterion to define the preferred sequence length. Molecular dynamic simulations with a 5-ns trajectory were performed using Amber 10 software and allowed the binding energies to be obtained. The binding modes of EFBA 8-mer and its SELEX analogue were determined. Binding energies were determined using the molecular mechanics Poisson–Boltzmann surface area/generalized Born surface area (MM-PBSA/GBSA) algorithms [52]. The binding energy between the thrombin and SELEX aptamers was higher than the energy between the thrombin and EFBA aptamers. Because of this issue, the use of the EFBA method is not encouraging.

Thrombin is one of the most popular objects in aptamer chemistry because of the peculiarities of its geometry and its production of high-affinity aptamers to two epitopes [53]. Thus, a series of DNA aptamers towards human thrombin was developed [27]. The tertiary structure of aptamers was predicted using PyMOL 1.1 and 3DNA [54]. The GROMACS 4.0 program package [55] and two force fields, parm99 [56] and parmbsc0 [57], were used to perform molecular dynamic simulations. MD was done using the isothermal-isobaric NPT ensemble and midrange trajectories from 60 to 200 ns. Using a previously known 15-mer G-quadruplex, a 29-mer and a 31-mer were designed.

Thrombin aptamer TBA15 and its modifications were studied in yet another study [28,29]. Thrombin-binding aptamer TBA15 has the 5′-GGTTGGTGTGGTTGG-3′ sequence. Chemical modification and the addition of a duplex module to the aptamer core structure were done to find whether chemical modification of the aptamer could improve its affinity to thrombin. The sequence of duplex-added TBA31 aptamer was 5′-CACTGGTAGGTTGGTGTGGTTGGGGCCAGTG-3′. The MD simulations were done using the Amber 8 program package along with ff99SB [58] and parmbsc0 [57] force fields. MD calculations were performed using the NPT ensemble while the trajectory length was up to 35 ns [28]. The MM-GBSA method was used to calculate the thrombin/aptamer-binding free energy. Novel triazole-modified and duplex-added aptamers showed pronounced thrombin-inhibiting activity. In another study by the same group of researchers, 5-nitoidole-modified TBA15 aptamer was studied, and it showed improved binding affinity to thrombin [59].

The Nanoscale Molecular Dynamics (NAMD) program [60] was used to perform the simulation with a CHARMM force field [61] for an 11-mer single-stranded DNA aptamer (PDB ID 2AVJ) and α-thrombin [30]. The sequence of this DNA was 5′-GGGGTTTGGGG-3′; it was used in a quadruplex form in MD analysis. This DNA aptamer had a TTT linker, which was responsible for forming the folds. It also provided the DNA its signature preferred loop conformation. As a whole, DNA-coated nanopore was investigated and showed advantages in protein detection. In yet another study, DNA aptamers were designed against thrombin using MD simulations [31]. The ff12SB force field [62], the NPT ensemble, and a 120-ns trajectory were used for the analysis. The in silico-designed aptamer demonstrated several times higher efficiency than did the aptamer that had undergone clinical trials earlier. 

8-Aryl-guanine bases were intercalated into the G-tetrad and central TGT loop of the thrombin-binding aptamer to define their influence on the antiparallel G-quadruplex-folding and thrombin-binding affinity [32]. The initial aptamer/thrombin structure was obtained from X-ray crystallography (PDB ID 4DII). Aryl-modified guanines were optimized at the B3LYP/6-31G(d,p) level of theory and were used for parametrization of the aptamer topology. Detailed MD analysis, with the parm14SB force field [62], on the modified DNA/thrombin complexes occurred with no constraints and a 20-ns trajectory under the NPT ensemble. With modification at G8 of the central TGT loop, the aptamers produced the most stable G-quadruplex topology and exhibited the highest protein-binding affinity. 

Thus, almost a decade of thrombin aptamer investigation and molecular design allowed the application of in vitro mutation, truncation, and chemical modification along with molecular modeling. This allowed improvement of the aptamer affinity to thrombin and brought multiple benefits in blood coagulation control.

### 2.2. Infection-Related Proteins

HIV proteins are popular targets of aptamer design. Thus, for example, interactions of HIV1 integrase with a DNA aptamer were investigated using rigid docking and MD [33]. The quadruplex aptamer 93del (Protein Data Bank ID 1Y8D) with the 5′-GGGGTGGGAGGAGGGT-3′ sequence was docked into a positively charged cavity of HIV1 integrase with the *Hex* program [63]. The electrostatic potential calculations of HIV1 integrase and aptamer structures were done. A 35-ns MD simulation under the NPT ensemble was performed for the aptamer/HIV1 integrase complex. The hydrogen bonds formed between the aptamer and HIV1 integrase by interacting with key amino acid residues disturbed HIV1 integrase interactions with DNA. In the next study, yet another anti-HIV aptamer, T30695 (PDB ID 2LE6) with a sequence of (GGGT)_4_ [34], was docked into the HIV1 integrase in a fashion similar to 93del. However, 93del had lower binding energy than T30695, which is due to the additional interactions made by single nucleotide loops. There were four single nucleotide thymine loops in T30695 and only two in 93del. 

Another HIV1 enzyme, reverse transcriptase (HIV1 RT), interaction with an aptamer was investigated using molecular dynamics [35]. The purpose of the study was to gain insight into the conformational dynamics of HIV1 RT and its aptamer. A 100-ns molecular dynamics trajectory using an ff99SB force field [58] was reported. The binding free energies of the HIV1 RT with the RNA aptamer T1.1 (5′-GGGAGAUUCGGUUUUCAGUCGGGAAAAACUGAA-3′ sequence) and the HIV1 RT with the DNA substrate were calculated using the g_mmpbsa tool [64]. The binding energy with the aptamer was higher than the binding energy with the DNA substrate. In yet another study, binding between the HIV1 RT and UCAA aptamer family was studied using a joint experimental-theoretical approach [36]. RNA aptamers were modeled in silico with the help of the NMR experimental spectra. The 2-D structure of the aptamers was derived from NMR spectroscopy and predicted from sequence by using the free energy-based approach in the Vfold2D program [65]. IsRNA [66] was used for coarse-grained MD; the top 10 conformations with the lowest energy were further used in protein/aptamer docking with the help of the MDockPP program [67]. The combination of in silico modeling and NMR spectroscopy allowed conclusions to be made about protein/RNA binding.

P. Kumar and A. Kumar designed aptamers against the influenza hemagglutinin using the Monte Carlo method [18]. They used a dataset of 98 oligonucleotides. During the QSAR Monte Carlo analysis, CORAL software was used [68]. Experimental pIC50 values (negative logarithm value calculated from the inhibitory activity of IC50 nM) were obtained from a previous publication [69]. The interesting point was that CORAL needed neither structure prediction nor docking to perform the analysis. The ssDNA nucleotide sequence was used in SMILES (simplified molecular input line entry specification) notation to evaluate the correlation between the structural parameters and pIC50 values. This research resulted in the design of nine new aptamers with pIC50 up to 9.86. Therefore, it was demonstrated that the QSAR Monte Carlo method is legitimate for the design of novel DNA aptamers.

In 2019, the outbreak of a novel coronavirus (SARS-CoV-2) reached a global pandemic level. Nevertheless, the mechanism of the coronavirus infection is not yet fully understood. Moreover, there are no common therapeutic agents and vaccines against this disease. High-binding affinity aptamers targeting the receptor-binding domain (RBD) of the SARS-CoV-2 spike glycoprotein were established [37] using SELEX in vitro experiments combined with in silico studies. SMART-Aptamer 2 [70] was used for the analysis of in vitro SELEX DNA pools. Moreover, molecular docking and MD were used for the study of aptamer/RBD binding. The K_d_ values of the two optimized RBD aptamers demonstrated their high affinity toward SARS-CoV-2. This makes the designed aptamers potent tools in SARS-CoV-2 diagnostics and antiviral therapeutics.

Bacterial surface proteins are also a popular target of aptamer design. Thus, a series of anti-hepatitis B surface antigen (HBsAg) aptamers was designed using docking and MD [38]. Three original SELEX aptamers with affinity to HBsAg were truncated into five short aptamers. The 2-D structures of the aptamers were obtained using Mfold, and the 3-D structures were determined with RNAComposer. Flexible docking was conducted. MD calculations with a 20-ns trajectory were performed using the CHARMM27 force field topology [71]. The affinities toward HBsAg were thoroughly investigated with an MM-PBSA algorithm. The results showed that truncated aptamers bind to HBsAg “a” determinant region (amino acid residues 99–169). Residues with the most effective interactions with all five aptamers were determined to be the active binding residues. 

Aptamers against *Streptoccocus agalactiae* surface protein (PDB ID 2XTL) were studied [39]. The authors tested a computational approach for the aptamer design to find an RNA with the highest affinity to the protein. A previously known 56-mer RNA sequence was truncated, and the secondary structure was predicted using Mfold. The 2-D geometries were transformed into 3-D structures using the 3dRNA 2.0 web server [72]. RNA aptamers were docked into the protein using AutoDock Vina, and then the binding affinities were determined. The best aptamer was a 40-mer with high predicted affinity to the target.

### 2.3. Cancer-Related Proteins

A 41-mer RNA aptamer was designed using prostate-specific membrane antigen (PSMA) as a target [40]. The rational in silico design of aptamers was done using tertiary structure prediction and protein/RNA docking. The goal of the study was to develop a new aptamer with improved affinity to PSMA and to find nucleobases responsible for aptamer–PSMA interaction. RNAstructure 4.6 [73] was used to predict the secondary structure of RNA aptamers. Using a “rational truncation” technique, nucleobases were removed from the 5′ and 3′ ends of previously known PSMA RNA aptamer to predict the secondary structure of the remaining sequence to be as similar as possible to the initial full-length aptamer. RNA 3-D structure minimization was done using the Amber program [74]. Molecular docking allowed the identification of key nucleobases critical for binding to PSMA and the suppression of its enzymatic activity. The binding sites of the aptamer on PSMA were globally searched by using the fast Fourier transform-based molecular docking program MDockPP [75].

EpCAM is a 40-kDa transmembrane glycoprotein that participates in cell adhesion, migration, proliferation, differentiation, and cellular signaling [76]; it is the predominant surface antigen in human colon cancer. EpCAM 15-mer RNA aptamers were designed and studied by Bavi et al. [41]. Secondary RNA structures were predicted using the RNA Vienna program [77]. The 2-D geometries were converted into the 3-D form using Rosetta software. The resulting geometries were docked into rigid EpCAM. The molecular dynamic simulation of the complexes obtained was done using the Amber 99SBildn force field [78]. A 40-ns MD trajectory was used in further analysis. The protocol for calculating the binding free energy according to MM-PBSA methodology was described previously [79] and was exploited to calculate the binding free energies for EpCAM/aptamer complexes.

Further, several EpCAM aptamers have been designed in silico [42]. The binding modes of aptamers were optimized with MD and docking. The RNA aptamer structure was predicted using Mfold. Then, MD simulations with a 450-ns trajectory were performed using NAMD 2 [60] and the ff14SB force field [80]. The CHARMM36 force field was used for EpCAM MD simulations. These calculations were followed by docking in Dot 2.0 [81]. MD simulations were performed on the 10 best aptamer conformations predicted by the docking. Experiments confirmed that the two best aptamers possessed affinities higher than the previously patented nanomolar aptamer EP23.

DNA aptamers were designed against carcinoembryonic antigen (CEA; PDB ID 2QSQ) [43]. A DNA mutant library was developed via nucleobase substitution or addition to the parent (P) sequence (5′-ATACCAGCTTATTCAATT-3′). The Mfold and RNAComposer web servers were used for secondary and tertiary structure prediction, respectively. Molecular docking was performed using ZDOCK [82], which searched all possible binding modes in the translational and rotational space between a DNA aptamer and a CEA protein and calculated each conformation using an energy-based shape complementary scoring function. The study showed that an in silico post-SELEX screening approach was feasible for improving DNA aptamers. The high affinity of the developed aptamers to CEA was confirmed experimentally.

Aptamers towards transmembrane glycoprotein mucin 1 (MUC1) were investigated [44]. One of the goals of the study was to identify mutants in the T11–T13 region that are capable of a tight interaction with the APDTRPAPG epitope. MD simulations were carried out to examine the intermolecular association between the MUC1 epitope APDTRPAPG and a series of aptamers derived from the structure of the parent S2.2 MUC1 aptamer (5′-CAGTTGATCCTTTGGATACCCTG-3′ sequence). MD simulations were performed with the Amber 16 program package. MM-GBSA binding free energy calculations were done to evaluate the strength of MUC1–aptamer interactions. MD, MM-GBSA, and conformational analysis revealed the novel MUC1 aptamer with mutations located at T11 and T12 residues. 

### 2.4. Other Proteins

**Figure 3:**
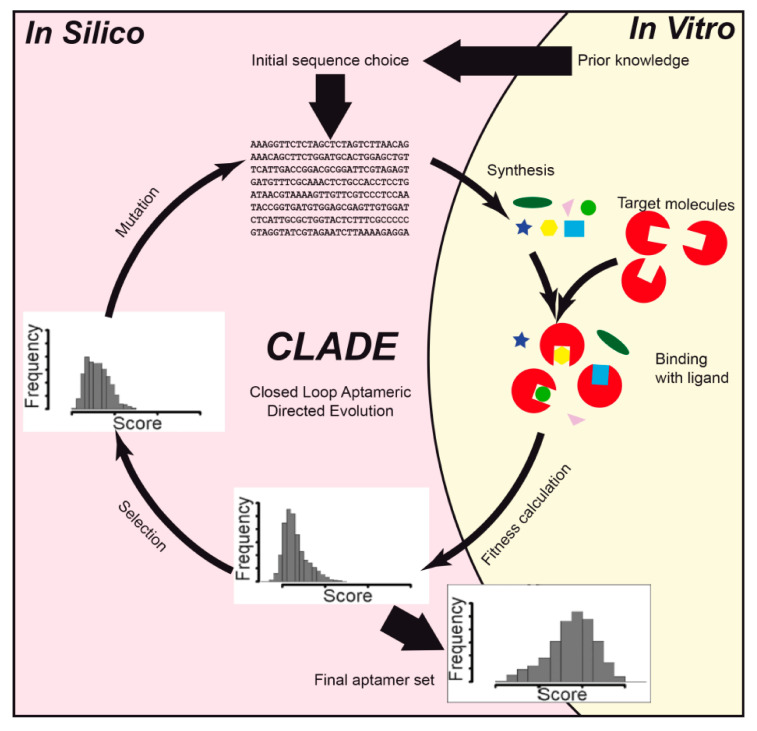
A scheme of a combined in silico and in vitro closed loop aptameric directed evolution (CLADE) approach.

Thus, Knight et al. [45] designed aptamers against a relatively large 110-kDa fluorescent protein allophycocyanin. The aim of the study was to apply machine learning to develop a novel method for the rapid design of aptamers with desired binding properties. A combined in vitro and in silico approach, called closed loop aptameric directed evolution (CLADE) (Figure 3), was applied. The ssDNA structure was predicted with UNAFold 3.4 software [83]. Statistical analysis was done using several statistical methods in R programming language. The random forest gave a high correlation coefficient score of 0.87 between in silico and in vitro binding.

Computational simulations of angiopoietin-2 (Ang2)–aptamer interactions were performed [46] by using ZDOCK and ZRANK docking functions in Discovery Studio 3.5. The purpose of the study was to introduce a computational approach to screen aptamers with high binding affinity to Ang2. Sixteen RNA aptamers were collected from previous studies and then truncated. The 2-D structures of the aptamers were generated with the CentroidFold web server (http://www.ncrna.org/centroidfold) [84]. The 3-D RNA structures were generated using the RNAComposer web server (http://rnacomposer.cs.put.poznan.pl) [85]. It was found that the aptamer with the highest target-binding affinity (5′-ACUAGCCUCAUCAGCUCAUGUGCCCCUCCGCCUGGAUCAC-3′ sequence) can bind Ang2 with a low K_d_ equal to 2.2 nM.

A series of five anti-Ang2 aptamers was designed using RNA structure prediction and molecular docking methods [47]. The goal of the study was to test a novel strategy to validate the reliability of the 3-D structures of aptamers produced in silico. The work consisted of three phases: (1) production of a large set of conformations for each aptamer, (2) rigid docking into the Ang2 protein, and (3) characterization of the Ang2/aptamer complexes. Thus, the SimRNA software [86] was used for tertiary structure prediction. The docking was performed using AutoDock Vina [87]. The calculated binding scores of the Ang2/aptamer complexes were based on the calculation of the “effective affinity”, which is the sum of the conformational energy (from SimRNA) and the docking energy (from AutoDock Vina). Effective affinities were in agreement with previous experimental studies.

Shcherbinin et al. [48] investigated and designed aptamers toward cytochrome p450. The aim of the study was to test a novel computational strategy and to find new cytochrome p450 aptamers. The study involved three phases: (1) finding a potential binding site, (2) designing novel aptamers, and (3) evaluating the experimental affinity. Thus, a series of 15-mer aptamers were designed using molecular docking and MD. Docking was performed using DOCK 6.5 [88]. During docking, amino acids and the structural part of the ligand were rigid, whereas nucleobases of the recognition part of the nucleic acid were flexible. The poses and conformations of oligonucleotides were analyzed using the SYBYL 8.1 package. The ff99SB force field was used for cytochrome, and the parmbsc0 was used for oligonucleotides. The binding free energies for the aptamer/protein complexes were calculated using MM-PBSA and normal mode analysis (NMA) methods implemented in Amber 9. MM-PBSA used ensembles obtained from MD simulations. Binding energy calculations consisted of the calculation of (1) interaction energy between cytochrome p450 and aptamer in the gas phase and (2) the solvation free energy. The gas phase interaction energy was the sum of van der Waals and electrostatic energies, whereas the solvation energy was the sum of nonpolar and polar energies. The stability of the complexes with cytochrome P450 was analyzed using MD simulation with a 10-ns trajectory under the NPT ensemble. 

RNA aptamers towards 66-kDa protein estrogen receptor alpha (ERα) were investigated [49]. The purpose of the study was to find an aptamer with the highest possible affinity to ERα. A series of 18 aptamers were designed and docked into ERα using AutoDock Vina, Haddock, and PatchDock [87,89,90]. The docking was flexible in AutoDock Vina and Haddock, whereas in PatchDock, the docking was rigid. The strength of binding in the ERα/RNA complexes, the intermolecular H-bonds, and hydrophobic interactions were measured. H-bonding dominated in the case of the best aptamer; its binding energy was equal to ΔG = −11.1 kcal mol^−1^. The resulting 17-mer had a sequence of 5′-GGGGUCAAGGUGACCCC-3′. Target specificity of the best aptamer was confirmed experimentally with cytochemistry and solid-phase immunoassays.

A series of four ssDNA aptamers was designed to inhibit the activity of angiotensin II [50] after preliminary SELEX experiments had been done. The aim of the study was to present a combined method that could be used to predict the 3-D structure of an ssDNA aptamer and its interaction with the protein. In silico analysis was done using online web servers. The Mfold 3.1 program (http://unafold.rna.albany.edu) [91] was used to predict the secondary structure of the aptamers, and RNAComposer (http://unafold.rna.albany.edu) was used to model the tertiary structure. Molecular docking was performed in ZDOCK 3.0 (http://zdock.umassmed.edu) [82]. The best aptamer had ΔG equal to −19.1 kcal mol^−1^. Protein–aptamer interactions were also analyzed experimentally.

Rabal and co-workers designed murine T-cell immunoglobulin mucin-3 (TIM-3) aptamers with both SELEX and in silico methods [51]. The tertiary structure was predicted using Rosetta [92]. Rigid docking of RNA aptamers into TIM-3 was done in the 3dRPC program [93]. Docking scoring parameters were analyzed along with experimental K_d_ values. Binding sites and binding modes in TIM-3/aptamer complexes were defined. 

One can see that a full set of molecular modeling methods, including secondary and tertiary structure prediction, docking, MD, and QSAR, can be employed in designing aptamers against protein targets. Several proteins were especially popular in these studies, for example, thrombin and HIV1 reverse transcriptase, because of their significant biomedical importance. This provides multiple benefits to researchers who begin to study these aptamers. The next section is dedicated to antibiotic aptamers, which are also a popular subject of in silico studies.

## 3. Antibiotics as Targets of Aptamer Design

A large pool of in silico aptamer design studies is dedicated to low-molecular-weight compounds as targets. This interest is largely associated with the possibilities of using aptamers in analytical systems. High stability, ease of regeneration, and relatively low cost make aptamers a promising alternative to antibodies, which are mainly used for these purposes at present [94].

These practically important low-molecular-weight targets can be divided into three classes, namely, antibiotics, organophosphates, and other compounds. This section will be dedicated to antibiotics as targets of aptamer design (Table 2). 

The common problem of in silico antibiotic aptamer design is in testing new methodology and finding new sequences with improved affinity to the target. Aminoglycosides are obviously the most popular targets of in silico aptamer modeling among antibiotics [1,22,95]. 

Chushak and Stone [1] designed RNA aptamers toward a set of antibiotics, namely, gentamicin, neomycin, and tobramycin. The primary aim of the study was to test a novel aptamer design strategy. RNA secondary structure generation was done in Vienna RNA web service [97]. The RNA geometries were optimized using the Amber 10 package and Amber99 force field. They studied 27-base RNA molecules with a 5′–GGC–N21–GUC-3′ sequence and a central random region of 21 nucleobases. The constant trinucleotides at the 5′-end and 3′-end relate to sequences of well-known theophylline aptamers. The developed aptamer selection technique was rather complicated and included two levels. At the first level, the following constraints were applied to the aptamers: (1) the free energy of the secondary structure formation was set to −5.7 kcal mol^−1^ or lower, (2) more than 10 nucleotides must not form Watson–Crick pairs, and (3) the number of the structures was limited to 150. At the second level, molecular docking was applied to identify aptamers that bind to the target. The selected RNA molecules were placed into a pool of sequences for experimental screening and selection of high-affinity aptamers. The docking was done in AutoDock 4.0 (autodock.scripps.edu). RNA aptamers were used as rigid molecules, and no metal ions were added during the docking. The predicted binding energies were in good agreement with experimental values. Thus, for example, the predicted gentamicin binding to the NMR-determined aptamer geometry (PDB ID 1BYJ) possessed −11.4 kcal mol^−1^, whereas the experimental value was equal to −10.9 kcal mol^−1^. 

Domin et al. [22] designed RNA riboswitches against tetracycline and streptomycin. RNAFold was used for the prediction of the secondary structure [77]. The aptamers were designed using randomly generated spacers, with a length from 6 to 20 bases, located between the antibiotic sensor sequence and the 3′-end part terminator. The resulting riboswitches contained a tetracycline aptamer “cb32sh” (in case of streptomycin “motif 1” aptamer). The design procedure consisted of a candidate generation and in silico minimal free energy calculation using RNAFold, which resulted in 25 tetracycline and 11 streptomycin riboswitch candidates. The activity of in silico-designed constructs was approved by in vitro experiments. 

The performance of two neomycin aptamers was compared [95], also considered the binding to other aminoglycosides. These NEO1A and NEO2A aptamers possessed a 43% sequence similarity. MD simulations were performed using the Amber99SB force field. Minimized aptamer structures were equilibrated under NVT and NPT ensembles at 298 K for 100 ps. A 20-ns MD simulation was performed with constant pressure and temperature. It was shown that the two neomycin-B aptamers show similar binding affinity, activity, and selectivity, despite structural differences.

A computational study of sulfadimethoxine aptamers was performed [96]. The purpose of their study was to test new computational methodology and to find a new aptamer with improved affinity to sulfadimethoxine with the application in aptasensor design. The native aptamer had a 5′-GAGGGCAACGAGTGTTTATAGA-3′ sequence. The tertiary structure of the aptamer was predicted by applying the Blast/PSI-Blast sequence-finding method [98]. The authors performed a step-by-step mutation of the native aptamer, based on MD calculations. MD was performed using the CHARMM27 force field. First, the aptamer MD simulation was done in the presence of sulfadimethoxine. The aptamer’s affinity to the target was determined through the calculation of binding Gibbs free energy using the MM-PBSA method. Then, a so-called conformational factor P_i_ [99] was evaluated as a measure of the target binding to each nucleotide of the aptamer. A nucleobase mutation was done for the residue with the smallest P_i_ value to the nucleobase with the highest P_i_ value, which led to the creation of a mutant sequence. MD simulation was done for the antibiotic/mutant complex, and the binding Gibbs energy and P_i_ were calculated again. The designing procedure was done repeatedly and resulted in the creation of five mutant aptamers with an improved affinity to sulfadimethoxine. The aptamer/target complexes were equilibrated in the NVT and NPT ensembles for 100 ps prior to 100-ns MD simulation. The described techniques improved the binding Gibbs energy from −24.9 kcal mol^−1^ for the native aptamer to −163.5 kcal mol^−1^ for the resulting 5′-AAGGGCAAGGAGGGTTCCTAGA-3′ sequence.

The calculation of conformational factor P_i_ and the subsequent mutation of nucleotides in the study by Khoshbin and Housaindokht [96] looks like a promising methodology of aptamer mutation and design.

## 4. Organophosphates as Targets of In Silico Aptamer Modeling

This section is dedicated to organophosphates as targets of aptamer design (Table 3). Some of the compounds regarded in this section are toxic substances, such as paraoxon. Others, such as free nucleotides (phosphorous ethers of nucleosides), are important biological compounds; they are structural elements of nucleic acids and coenzymes. For this reason, the popularity of mononucleotides as targets of aptamer design is explicable. 

In one of the pioneer reports relating to in silico aptamer design [100], guanosine triphosphate (GTP) was used as a target. Enrichment of in vitro SELEX aptamer pools with complex aptamers can extend the probability of discovering new aptamers. An approach for designing RNA pools was also reported. The web server RAGPOOLS (RNA-As-Graph-Pools) was developed for the design and analysis of structured aptamer pools for SELEX (http://rubin2.biomath.nyu.edu/home.html). 

Chushak and Stone [1] designed RNA aptamers toward a large set of compounds, which was discussed in the previous section. The aim of this study was to test the aptamer design methodology on multiple target compounds. They designed aptamers against mononucleotides FMN and ATP. They used docking to make binding energy predictions. Both 35- and 40-base RNA aptamers were designed toward FMN and ATP, respectively. In silico-predicted binding energies for FMN/aptamer and ATP/aptamer complexes were in agreement with the experiment.

Bioinformatic tools for ATP aptamer many-way junction creation were developed [101]. It is well known that RNA/DNA aptamers obtained using SELEX are not structurally diverse and mostly consist of simple topological geometries, such as stem loops; for this reason, many-way junctions are not frequent in ATP aptamers. The structural variety of the starting RNA/DNA pool can increase the probability of finding new aptamers with improved affinity [106]. Two methods of structural complexity and diversity improvement were developed: random filtering (RF) and genetic filtering (GF). RF starts from a random aptamer pool and calculates the number of junctions for each nucleotide sequence in the pool. Each 5-way junction sequence is then mutated at every single-stranded site one million times to evaluate the structural distribution of the respective RNA/DNA pool design. The pool with the highest percentage of five-way junctions was selected. Using the Vienna RNA program to fold a million 100-base random sequences, 76 5-way junction sequences were defined. These sequences were subjected to RF to create a 5-way junction-enhanced pool. The idea of GF is similar; its purpose was to create a pool of 1-way, 2-way, 3-way, 4-way, and 5-way junctions with an equal distribution of 20% each. A pool designed with GF was synthesized and subjected to a SELEX experiment for ATP aptamers. After eight rounds of selection, complex five-way junction topologies still accounted for a sizable percentage of the pool, confirming that RF and GF greatly improved generation of high-complexity structures and that these structures were maintained during the selection process. As a result, these techniques seem to be promising because one of the obtained five-way junction aptamers demonstrated improved K_d_ values compared to previously published ATP aptamers: 3.7 and 6.0 μM, respectively.

Tseng et al. designed a ssDNA aptamer towards cell membrane phospholipid phosphatidylserine (PS) using the entropic fragment-based approach (EFBA) [28]. The approbation of EFBA methodology was a primary aim of the study. Both GAMESS US and AM1 semi-empirical methods were used to optimize PS geometry prior to molecular dynamic simulation. EFBA was used to determine the nucleobase sequences that most likely interact with the target molecule. Molecular dynamic simulations allowed binding energies to be obtained. The experimentally determined binding energy between the PS and EFBA six-nucleotide DNA aptamer was low. This obstacle makes the EFBA technique less than promising.

In yet another investigation, the EFBA algorithm was applied to design a DNA aptamer that binds specifically to PS [102]. PS geometry was taken from the previous study [28]. MD simulations were performed using an ff03 force field [107] with a constant temperature of 300 K and a 10-ns trajectory. The binding energies for aptamer/PS complexes were determined both computationally and experimentally. This study identified a short 6-mer oligonucleotide sequence as a prospective ssDNA aptamer for PS detection.

Jokar and co-authors designed ssDNA aptamers against the widely known organophosphorus insecticide diazinon [103]. The aim of the study was to develop a biosensor for diazinon detection. The docking of diazinon into aptamers was performed using the Lamarckian genetic algorithm (LGA) for flexible ligand/receptor docking. Docking revealed that one of the most influential factors in the stability of the aptamer/diazinon complex was the number of H-bonds. V-rescale coupling was used to maintain constant temperature and pressure during MD simulations. Prior to MD simulation, equilibration under the NPT ensemble was used for 200 ps. Major MD calculations occurred under the NVT ensemble with a 10-ns trajectory; CHARMM22 and CHARMM27 force fields were used for the aptamers and diazinon, respectively. The QGRS Mapper server [108] indicated that only one ssDNA sequence was able to form G-quadruplexes. This G-quadruplex DNA demonstrated that it is a reliable candidate for diazinon sensing both theoretically and experimentally, once again indicating that aptamers with complicated topology are favorable for high affinity with targets. 

Immobilization of the FMN aptamer on a gold surface was studied [104]. The aim of the research was not to describe the aptasensor but rather to evaluate the interaction energy between RNA and FMN using the MM-GBSA algorithm. MD calculations were performed with a force field composed of the ff99SBildn [109] and gaff [110] set of parameters to describe the RNA system interaction with FMN. MD simulation of the RNA/FMN complex was conducted under the NPT ensemble with a 100-ns trajectory. The ligand/aptamer binding increased significantly (from −24.6 to −11.6 kcal mol^−1^) when the system was immobilized on a gold surface, which was in agreement with the experimental data.

In the next study, paraoxon aptamers were designed [105]. The goal of the study was to develop an approach to rational in silico design of aptamers for organophosphates using paraoxon as an example. The 3-D structure of a previously known 35-nucleobase aptamer (5′-AGCTTGCTGCAGCGATTCTTGATCGCCACAGAGCT-3′ sequence) was predicted using HyperChem software and then was optimized with a 10-ns MD and energy minimization. Using the Discovery Studio program, 3-D aptamer structure mutation was performed. Binding energy ΔG and dissociation constant K_d_ were evaluated using the Lamarckian genetic algorithm [111]. For each aptamer/ligand complex, 50 conformations were achieved. The conformation with the lowest K_d_ was selected as the terminal one and was used as the starting geometry for further MD simulations. A constant temperature (300 K) and pressure (1 bar) were maintained with a V-rescale thermostat and a Berendsen barostat. Before running the main MD simulations, 100-ps equilibration was performed. MD with a 10-ns trajectory was performed using the MM-PBSA method to calculate the aptamer/paraoxon complex binding energy. Only the T17C mutation allowed for the improvement of the affinity between the aptamer and ligand (from −31.0 to −32.3 kcal mol^−1^), whereas mutations at positions 18, 19, and 20 did not allow for any increase of the affinity. The double mutation T17C-C18T also increased the effectiveness of ligand binding (−32.8 kcal mol^−1^). Thus, the aptamers with improved affinity to paraoxon were designed using only in silico approaches.

## 5. Different Low-Molecular-Weight Compounds as Targets of Aptamer Design

This section is dedicated to other targets—low-molecular-weight compounds neither antibiotics, nor organophosphates (Table 4). The typical aim of the studies in this section is the approbation of in silico aptamer design methodology and to verify that the improvement of aptamer affinity toward target molecules is not rare. The popular low-molecular-weight targets include nucleobases, amino acids, amino acid derivatives, therapeutical agents, and hormones.

RNA aptamers were designed toward multiple compounds by Chushak and Stone [1], whose work we reviewed in previous sections. Among other targets, they designed aptamers against arginine, codeine, guanine, isoleucine, and theophylline. They used rigid docking to make binding energy predictions. In most cases, the experimental and predicted binding energies were similar. For example, the experimental binding energy between guanine and its aptamer was equal to −7.8 kcal mol^−1^, and the in silico value was equal to −7.7 kcal mol^−1^; for isoleucine, the experimental binding energy was equal to −4.0 kcal mol^−1^, and the in silico value was equal to −4.2 kcal mol^−1^.

Lin with co-authors studied DNA aptamer (sequence 5′-GATCGAAACGTAGCGCCTTCGATC-3′) binding with argininamide (Arm) [112]. The aim of the research was to examine critical nucleotides involved in aptamer/Arm binding. MD simulations were performed using the NPT ensemble with a 20-ns trajectory and the Amber94 force field. The initial complex geometry was taken from PDB (PDB ID 1OLD). The nucleotides C9, A12, C17, and Watson–Crick pair G10-C16 were important for the aptamer/ligand binding. The critical nucleotides in DNA/Arm binding provided valuable information for further DNA aptamer design.

In the next study, the binding characteristics of a DNA aptamer to Arm and other arginine-like targets were investigated [113]. The aim of the study was to provide valuable guidelines for the application of docking methodology and the prediction of aptamer/ligand binding energies. Docking was performed on seven NMR-obtained aptamer geometries (PDB ID 1OLD). The Lamarckian genetic algorithm (LGA) and Amber03 force field [57] were used. Global docking was done. Docking simulation was performed on the entire DNA geometry, and the geometry of the ligands was flexible, whereas the aptamer was rigid. The best docking poses were determined after an energy minimization of Arm/DNA complexes. Experiments reflected that D-Arm binds slightly stronger to the aptamer than does L-Arm; K_d_ was equal to 135 μM and 98 μM for L-Arm and D-Arm, respectively. This fact was partially confirmed by docking; the calculated K_d_ was equal to 343 and 643 pM for D-Arm and L-Arm, respectively, whereas the binding energies were −12.1 and −11.6 kcal mol^−1^, respectively. L-Arg and D-Arg possessed significantly lower affinities toward the aptamer, which was evidenced both experimentally and computationally. The inability of L-lysine to bind to the DNA was computationally confirmed by low binding affinity. Therefore, theoretically defined binding energies of Arg-like ligands showed a good correlation with the experimentally evaluated binding energies.

L-Arm dsDNA aptamers with imidazole-tethered thymines were investigated using molecular dynamics [114]. The goal of the study was to investigate the influence of chemical modification on aptamer/ligand binding. The initial dsDNA geometry was predicted in Discovery Studio 4.0. Aptamer/dsDNA complexes were heated to 300 K, using the Langevin temperature equilibration protocol, during a 20-ps NVT run. Additionally, the system was equilibrated for 100 ps under the NPT ensemble prior to using a 50-ns MD simulation under the NPT ensemble. These MD simulations were accompanied by UV spectroscopy and NMR. It was demonstrated that thermal stabilizing effects occur upon addition of a single imidazole-tethered thymidine; the hydrogen bond forms between imidazolium residue and the Hoogsteen side of a guanosine residue on the opposite DNA strand. Moreover, multiple imidazolium moieties also increase the thermal stability of the DNA aptamer.

Using the well-known theophylline aptamer as a sensor, the actuator part of the riboswitch was designed [115]. The aim was to design a riboswitch capable of performing ligand-dependent control of *E. coli* gene expression. The designed riboswitches consisted of four parts: the theophylline aptamer, a 6- to 20-base spacer, a sequence complementary to the 3′-end of the aptamer (3′-part terminator), and a poly-U stretch at the 5′-end. The RNA secondary structure prediction and free energy evaluation were done. The energy difference between the free energy of the folded full-length riboswitch and an aptamer constrained to form the ligand-binding complex was calculated. A total of six riboswitches were experimentally tested. The riboswitch with the lowest free energy value produced a stable structure that could not be disturbed on theophylline binding; for this reason, this construct was always in an OFF state. For other structures, equilibrium was shifted toward the aptamer/ligand binding (always in an ON state). The functional riboswitches are the golden mean; they demonstrated intermediate stability and allowed the desired ligand-dependent rearrangement of the constructs, switching between ON and OFF states. As a result, several riboswitches showed ligand-dependent control of gene expression in *E. coli*, demonstrating that it is possible to design riboswitches for transcription regulation.

The aim of the study by Zhou et al. was to test a so-called in silico SELEX approach for the theophylline aptamer design [116]. The approach consisted of two phases. First, secondary structure-based sequence screening occurred, whose purpose was to select the sequences that can form an acceptable RNA motif. Second, sequence enrichment regarding theophylline binding by MD was virtually screened. The original theophylline/RNA complex was obtained from PDB (PDB ID 1O15). The x3DNA program was used to make in silico mutations. The final round of MD with a 100-ns trajectory included 24 RNA sequences. Binding energies of the target/aptamer complexes were calculated using the MM-PBSA algorithm. Six potent aptamers, which were derived from a space containing 413 sequences, were experimentally determined to bind the theophylline with high affinity (see Table 4 for details). These results demonstrated the high potential of in silico SELEX as a method for aptamer design and optimization.

The aim of Jokar et al. was to design an ssDNA-based aptasensor for the detection of the insecticide acetamiprid [117]. Docking was performed in AutoDock, which revealed two loops as active sites in the aptamer. Circular dichroism spectroscopy and colorimetry confirmed aptamer folding due to stem-loop formation upon acetamiprid binding. Stability of the DNA/ligand complex was demonstrated by MD simulations with a 100-ns trajectory.

Tomita and co-authors designed aptamer sensors based on a microarray assay that was combined with the computational secondary structure prediction [118]. The aim was to optimize the patulin aptamer. This was performed using in silico structure prediction. Three rules were applied to the in silico aptamer selection (see Table 4). The microarray aptamer library included 10^4^ sequences, whereas the in silico library consisted of more than 800,000 structures. As a result of this joint theoretical-experimental research, a new patulin aptamer was designed.

Hilder and Hodgkiss designed DNA aptamers towards 17β-estradiol (E2) [119]. The 2-D aptamer structures were determined using Mfold or RNAstructure, and, subsequently, the 3-D structures were defined using RNAComposer. Rigid docking of aptamers to E2 was performed. The stability of the top-ranked complexes was checked by MD with a 30-ns trajectory. A CHARMM36 force field was used along with the CHARMM27 for DNA aptamer structures. The free energy of binding was calculated using the free energy perturbation (FEP) method, which resulted in excellent agreement between the computations and the experiment for the best E2 aptamer (5′-GCCGTTTGGGCCCAAGTTCGGC-3′ sequence); the computational K_d_ value was equal to 10.9 nM, and the experimental value was 11 nM. 

Zhao et al. investigated N-butanoyl-L-homoserine lactone (C4-HSL) aptamers using SELEX along with in silico tertiary structure prediction [120]. The purpose was to screen DNA aptamers against C4-HSL for the inhibition of biofilm formation of *Pseudomonas aeruginosa*. The 3-D RNA structure prediction showed that the designed aptamers possessed a highly conserved Y-shaped structural unit, which is probably responsible for C4-HSL binding. In vitro biofilm inhibition experiments showed that the activity of *P. aeruginosa* was efficiently reduced to about one-third by the aptamers.

## 6. Conclusions

In this study, we reviewed theoretical and joint theoretical-experimental studies on DNA and RNA aptamers. We regarded several classes of aptamer targets, namely, proteins; antibiotics; organophosphates; and other low-molecular-weight compounds, which included nucleobases, amino acids, and drugs. During aptamer modeling and in silico design, a full set of molecular modeling methods might be used, namely, docking, molecular dynamics, quantum-chemical calculations, and even the quantitative structure–activity relationship (QSAR). Only a few of the developments carried out to date have used the QM/MM method, which can give fruitful results in the analysis of the structural patterns of the aptamer/ligand complex. Additionally, the application of the QSAR technique is very rare; only one found paper described its use in aptamer designing [18]. QSAR and machine learning can provide serious benefits for aptamer design and modeling.

To date, the methods of computational analysis of the structure and properties of aptamers have shown their effectiveness. The in silico predictions are confirmed in vitro and make it possible to increase the affinity of aptamers in relation to their target analytes, as well as to ensure the stabilization of required conformations. In the framework of further work, the integral application of considered here methods of in silico design is in demand for efficient control of the binding properties of aptamers in different reaction media, changes in their cross-reactivity with respect to structurally related compounds, construction of oligonucleotide chains uniting several spatially close binding sites for multispecific aptamers, and also for regulators of the binding properties of aptamers.

## Figures and Tables

**Table 1:** Main features of aptamer design studies in which proteins were used as the target.

Source	Target	Computational Methods	Software	Description
[26]	Thrombin	Structure prediction, molecular dynamics (MD)	3D-DART, Amber 10	Molecular dynamics along with entropic fragment-based approach (EFBA) allowed designing a DNA aptamer, which was surpassed by an aptamer obtained using SELEX prior to it.
[27]	Thrombin	Structure prediction, MD	PyMOL 1.1, 3DNA, GROMACS 4.0	In silico calculations were accompanied by an in vitro thrombin inhibition assay. Two new thrombin aptamers, a 29-mer and a 31-mer with high inhibitory activity, were obtained.
[28,29]	Thrombin	MD	Amber 8	Novel triazole-modified and duplex-added aptamers showed potent thrombin-inhibiting activity.
[30]	Thrombin	MD	NAMD	DNA-coated nanopore for protein detection was investigated.
[31]	Thrombin	MD	Amber	The in silico-designed aptamer demonstrated seven times higher efficiency than previously known anti-thrombin aptamers.
[32]	Thrombin	MD	Amber 12	It was shown that the internal 8-aryl-guanine modification can manipulate the interactions between the DNA bases and the amino acid residues of thrombin. Nevertheless, guanine arylation at the G-tetrad reduced thrombin-binding affinity.
[33]	HIV1 integrase	Docking, MD	Hex, GROMACS	MD simulation was performed for the 93del/HIV1 integrase complex. HIV1 integrase interactions with the aptamer inhibited HIV1 integrase interactions with DNA.
[34]	HIV1 integrase	MD	Amber	Molecular dynamics were accompanied by nuclear magnetic resonance (NMR) spectroscopy and circular dichroism experiments. T30695 aptamer had a higher interaction energy (−116.4 kcal mol^−1^) than the previously known 93del aptamer (−103.4 kcal mol^−1^).
[35]	HIV1 reverse transcriptase (HIV1 RT)	MD	GROMACS 4.5	T1.1 RNA aptamer complex with HIV1 RT was more stable than that with a DNA substrate.
[36]	HIV1 RT	Structure prediction, docking, MD	Vfold2D, IsRNA, MDockPR, NAMD	The combination of in silico modeling and NMR allowed the identification of structural RNA elements critical for HIV1 RT inhibition and the determination of the role of UCAA motif in RT–aptamer interaction.
[18]	Influenza hemagglutinin	QSAR	CORAL	Experimental pIC50 values were used as a target parameter during QSAR modeling. The study resulted in the design of nine new aptamers with high inhibitory activity.
[37]	SARS-CoV-2 spike glycoprotein	Structure prediction, docking, MD	SMART-Aptamer 2, MFold, RNAComposer	Two potent and selective DNA aptamers were designed with equilibrium dissociation constant (K_d_) values of 5.8 and 19.9 nM.
[38]	Hepatitis B surface antigen (HBsAg)	Structure prediction, docking, MD	Mfold, RNAComposer, AutoDock Vina, GROMACS 5.1	It was determined that HBsAg/aptamer interactions were stabilized by the dynamic hydrogen bond formation between the active amino acid residues (“a” determinant region) and nucleotides.
[39]	*Streptococcus agalactiae* surface protein	Structure prediction, docking	Mfold, 3dRNA 2.0, AutoDock Vina	All seven RNA aptamers designed carried a hairpin. The best aptamer was a 40-mer with predicted ΔG equal to −14.7 kcal mol^−1^ and predicted affinity equal to −16.3 kcal mol^−1^.
[40]	Prostate-specific membrane antigen (PSMA)	Structural prediction, docking	RNAstructure 4.6, Amber, MDockPP	Using the “rational truncation” technique, bases were removed from the aptamer to predict the secondary structure of the remaining oligonucleotide. Molecular docking allowed the identification of binding sites of the aptamers on PSMA.
[41]	Epithelial adhesion molecule (EpCAM)	Structure prediction, docking, MD	Vienna RNA, Rosetta, AutoDock Vina, GROMACS 5.0	Flow cytometry and fluorescence microscopy showed that in silico-designed RNA aptamer interacts specifically with the cells that express EpCAM but not with the EpCAM-negative cells.
[42]	EpCAM	Structure prediction, docking, MD	Mfold, Dot 2.0, NAMD 2	The binding modes of aptamers were first predicted and then optimized with MD and docking. Titration calorimetry experiments confirmed that the designed aptamers possessed high affinity to EpCAM.
[43]	Carcinoembryonic antigen (CEA)	Structure prediction, docking	Mfold, RNAComposer, ZDOCK	According to ZDOCK, parent sequence with ATG attached to the 3′-end and GAC sequence attached to the 5′-end had the highest score among the designed aptamers. The high affinity of the developed aptamers was confirmed experimentally by bilayer interferometry.
[44]	Transmembrane glycoprotein mucin 1 (MUC1)	Docking, MD	AutoDock Vina, Amber 16	MD, molecular mechanics Generalized Born surface area (MM-GBSA), and conformational analysis revealed novel anti-MUC1 aptamer that might be used in anti-cancer therapy.
[45]	Allophycocyanin	Structure prediction, statistical analysis	UNAFold 3.4, R	A joint theoretical-experimental approach, called closed loop aptameric directed evolution (CLADE), was used when 44,131 aptamers were analyzed using the DNA microarray technique. Statistical analysis was done using random forest, regression tree, and genetic programming.
[46]	Angiopoietin-2 (Ang2)	Structure prediction, docking	Centroid-Fold, RNAComposer, Discovery Studio 3.5	Surface plasmon resonance along with Zrank algorithm realized in DS 3.5 allowed finding an RNA aptamer with high target-binding affinity.
[47]	Ang2	Structure prediction, docking	SimRNA, AutoDock Vina	The calculated effective affinities of the Ang2/aptamer complexes were in agreement with the experiment.
[48]	Cytochrome p450	Docking, molecular dynamics	DOCK 6.5, SYBYL 8.1, Amber 9	A series of aptamers was designed and showed selective affinity toward cytochrome p450.
[49]	Estrogen receptor alpha (ERα)	Docking	AutoDock Vina, Haddock, PatchDock	The aptamer was designed based on independent docking analysis in three different programs and was validated by measuring the thermodynamic parameters of ERα/aptamer interactions using isothermal titration calorimetry.
[50]	Angiotensin II	Structure prediction, docking	Mfold 3.1, RNAComposer, ZDOCK 3.0	The interactions of the aptamers with the protein were analyzed by means of surface plasmon resonance spectroscopy and were consistent with in silico data.
[51]	T-cell immunoglobulin mucin-3 (TIM-3)	Structure prediction, docking	RNAstructure 5.3, Rosetta, 3dRPC	Docking scoring parameters were analyzed along with experimental data. Binding sites and binding modes in protein/aptamer complexes were identified.

Abbreviations: angiopoietin-2 (Ang2); carcinoembryonic antigen (CEA); closed loop aptameric directed evolution (CLADE); dissociation constant (Kd); entropic fragment–based approach (EFBA); epithelial adhesion molecule (EpCAM); estrogen receptor alpha (ERα); molecular dynamics (MD); molecular mechanics Generalized Born surface area analysis (MM-GBSA); Hepatitis B surface antigen (HBsAg); HIV1 reverse transcriptase (HIV1 RT); nuclear magnetic resonance (NMR); prostate-specific membrane antigen (PSMA); T-cell immunoglobulin mucin-3 (TIM-3); transmembrane glycoprotein mucin 1 (MUC1).

**Table 2:** Main features of aptamer design studies in which antibiotics were used as the target.

Source	Target	Computational Methods	Software	Description
[1]	Gentamicin, neomycin, tobramycin	Structure prediction, docking	Vienna RNA, Rosetta, Amber 10, AutoDock 4.0	The procedure for the selection of aptamers was rather complicated and included the free energy of secondary structure formation calculation, RNA geometry optimization, and rigid docking. The predicted binding energies were in good agreement with experimental values.
[22]	Tetracycline, streptomycin	Structure prediction	RNAFold	Riboswitches were designed using randomly generated spacers with a length from 6 to 20 bases. The in silico design was based on a minimal free energy calculation, which consisted of an antibiotic aptamer, a spacer, a complementary part for the aptamer, and a poly-U sequence at the 3′-end. In the presence of tetracycline, the expression of β-galactosidase was induced in *E. coli*, resulting in the increase of the enzyme’s activity.
[95]	Neomycin-B	Structure prediction, MD	Mfold, GROMACS	Experimental NMR and titration colorimetry studies combined with MD simulations revealed that, despite the difference in nucleotide sequence, the structural and dynamical features of the studied aptamers were similar. The affinity of the aptamers toward other aminoglycosides was shown to be lower compared to the target.
[96]	Sulfadimethoxine	Structure prediction, MD	PSI-Blast, GROMACS 5.1	The aptamer’s affinity to the target was determined through the calculation of binding Gibbs free energy using the MM-PBSA method. The designing procedure was done repeatedly and resulted in a creation of mutant aptamers with the improved affinity to sulfadimethoxine.

**Table 3:** Main features of aptamer design studies in which organophosphates were used as the target.

Source	Target	Computational Methods	Software	Description
[100]	Guanosine triphosphate (GTP)	Graph theory, matrix analysis	RAGPOOLS	An approach for engineering RNA pools used an exact set of starting sequences and certain mutation ratios in specific locations within a random region. To produce these key parameters, graph theory and matrix analysis were used. The initial aptamer pools acquired by the described methodology provided the selection of RNAs with higher affinity when compared to the in vitro pools.
[1]	Adenosine triphosphate (ATP), flavin mononucleotide (FMN)	Structure prediction, docking	Vienna RNA, Rosetta, Amber 10, AutoDock 4.0	Both 35- and 40-base RNA aptamers were designed toward FMN and ATP, respectively. The in silico-predicted binding energy of, for example, FMN was in agreement with the experimental binding energy, −7.7 kcal mol^−1^ and −8.6 kcal mol^−1^, respectively.
[101]	ATP	Structure prediction	Vienna RNA, Mfold	Two methods of improvement of RNA/DNA aptamer complexity were created: random filtering and genetic filtering. One of the obtained 5-way junction aptamers demonstrated improved K_d_ values compared to those of native ATP aptamers.
[28]	Phosphatidylserine (PS)	Molecular dynamics (MD)	Amber 10	Molecular dynamics along with entropic fragment–based approach (EFBA) allowed designing a DNA 6-mer, which, however, possessed low binding energy.
[102]	PS	MD	Amber 11	The EFBA algorithm was applied to design a DNA aptamer that binds specifically to PS. This study identified the 5′-AAAGAC-3′ sequence as a prospective ssDNA aptamer for PS detection.
[103]	Diazinon	Structure prediction, docking, MD	Mfold, RNAComposer, AutoDock 4.2, GROMACS 4.5	Flexible ligand/receptor docking along with MD calculations under the NVT ensemble (10-ns trajectory) showed that G-quadruplex–forming aptamer is reliable for diazinon sensing.
[104]	FMN	MD	Amber 12	The binding energy of FMN/RNA complex was evaluated using MM-GBSA. FMN/aptamer binding increased significantly when the system was immobilized on the surface of gold, which is in accordance with the experimental data.
[105]	Paraoxon	Structure prediction, docking, MD	HyperChem, Discovery Studio, AutoDock 4.2, GROMACS 5.0	The T17C mutation allowed the improvement of the affinity between aptamer and ligand (from −31.0 kcal mol^−1^ to −32.3 kcal mol^−1^), and the T17C-C18T double mutation increased the effectiveness of ligand binding (−32.8 kcal mol^−1^).

**Table 4:** Main features of aptamer design studies in which low-molecular-weight compounds were used as the target.

Source	Target	Computational Methods	Software	Description
[1]	Arginine, codeine, guanine, isoleucine, theophylline	Structure prediction, docking	Vienna RNA, Rosetta, Amber 10, AutoDock 4.0	Rigid docking binding energy predictions were in good agreement with experimental values, which confirms good performance of the applied aptamer design methodology.
[112]	L-Argininamide (L-Arm)	MD	NAMD 2.6	G10, C16, C9, A12, and C17 bases were significant for aptamer/L-Arm binding, which is important for further aptamer design.
[113]	L-Arm, D-Arm, L-Arg, D-Arg, agmatine, ethyl-guanidine, L-Lys, N-methyl L-Arg	Docking	AutoDock 4.0	The interaction of eight arginine (Arg) like ligands with a DNA aptamer was analyzed. D-Arm possessed the highest affinity toward the aptamer. Theoretically defined binding energies and the K_d_ of ligands were in good agreement with experimentally determined values.
[114]	L-Arm	Structure prediction, MD	Discovery Studio 4.0, Amber 12	MD simulations of 50 ns were accompanied with UV spectroscopy and NMR. Thermal stabilizing effects occurred upon addition of the imidazole-tethered thymidines. Multiple imidazole moieties also maintained L-Arm binding capacity, which enhanced aptamer efficacy.
[115]	Theophylline	Structure prediction	RNAFold 2.0	The energy difference between the free energy of a riboswitch and a ligand-free aptamer was calculated. Several riboswitches were experimentally tested, and some of them showed ligand-dependent control of gene expression in *E. coli*, demonstrating that it is possible to design riboswitches for transcription regulation.
[116]	Theophylline	Structure prediction, MD	X3DNA, GROMACS 4.5	Six potent aptamers designed in silico were experimentally determined to bind theophylline with high affinity: K_d_ was equal to 0.16–0.52 μM, whereas K_d_ of the original theophylline/RNA complex was equal to 0.32 μM.
[117]	Acetamiprid	Structure prediction, docking, MD	Mfold, RNA Composer, AutoDock, NAMD 2.9	A DNA-based aptasensor was designed for the detection of acetamiprid. Docking revealed two loops as active sites in the aptamer. Circular dichroism spectroscopy and colorimetry confirmed aptamer folding due to stem-loop formation upon acetamiprid binding.
[118]	Patulin	Structure prediction	UNAFold 3.8	Microarray aptamer analysis was combined with in silico secondary structure prediction. In silico studies applied three conditions to the aptamers: (1) presence of a predicted secondary DNA structure producing one hairpin loop without a ligand, (2) hairpin loop with a length from 3 to 7 bases, and (3) stem length from 6 to 9 bases. As a result, a novel patulin aptamer was optimized.
[119]	17β-estradiol (E2)	Structure prediction, docking, MD	Mfold, RNAstructure, ZDOCK, RNAComposer, NAMD 2.10	Rigid docking of aptamers to E2 was used along with a 30-ns MD. It was demonstrated that E2 binds to a thymine loop region common to all E2-specific aptamers.
[120]	N-butanoyl-L-homoserine lactone (C4-HSL)	Structure prediction	RNAstructure 5.6, 3dRNA	The 2-D and 3-D RNA structure predictions showed that SELEX-designed aptamers possessed a conservative Y-shaped structural unit, which is probably responsible for C4-HSL binding.

## References

[1] Chushak Y., Stone M.O. (2009). In silico selection of RNA aptamers. Nucleic Acids Res..

[2] Kruspe S., Mittelberger F., Szameit K., Hahn U. (2014). Aptamers as drug delivery vehicles. ChemMedChem.

[3] Cai S., Yan J., Xiong H., Liu Y., Peng D., Liu Z. (2018). Investigations on the interface of nucleic acid aptamers and binding targets. Analyst.

[4] Yuce M., Kurt H. (2017). How to make nanobiosensors: Surface modification and characterisation of nanomaterials for biosensing applications. RSC Adv..

[5] Ren Q., Ga L., Lu Z., Ai J., Wang T. (2020). Aptamer-functionalized nanomaterials for biological applications. Mater. Chem. Front..

[6] Villalonga A., Pérez-Calabuig A.M., Villalonga R. (2020). Electrochemical biosensors based on nucleic acid aptamers. Anal. Bioanal. Chem..

[7] Tuerk C., Gold L. (1990). Systematic evolution of ligands by exponential enrichment: RNA ligands to bacteriophage T4 DNA polymerase. Science.

[8] Zhuo Z., Yu Y., Wang M., Li J., Zhang Z., Liu J., Wu X., Lu A., Zhang G., Zhang B. (2017). Recent advances in SELEX technology and aptamer applications in biomedicine. Int. J. Mol. Sci..

[9] Komarova N., Kuznetsov A. (2019). Inside the black box: What makes SELEX better?. Molecules.

[10] Bayat P., Nosrati R., Alibolandi M., Rafatpanah H., Abnous K., Khedri M., Ramezani M. (2018). SELEX methods on the road to protein targeting with nucleic acid aptamers. Biochimie.

[11] Antipova O.M., Zavyalova E.G., Golovin A.V., Pavlova G.V., Kopylov A.M., Reshetnikov R.V. (2018). Advances in the application of modified nucleotides in SELEX technology. Biochemistry.

[12] Hoinka J., Przytycka T. (2016). AptaPLEX—A dedicated, multithreaded demultiplexer for HT-SELEX data. Methods.

[13] McKeague M., Wong R.S., Smolke C.D. (2016). Opportunities in the design and application of RNA for gene expression control. Nucleic Acids Res..

[14] Hamada M. (2018). In silico approaches to RNA aptamer design. Biochimie.

[15] Emami N., Pakchin P.S., Ferdousi R. (2020). Computational predictive approaches for interaction and structure of aptamers. J. Theor. Biol..

[16] Yan Z., Wang J. (2017). SPA-LN: A scoring function of ligand-nucleic acid interactions via optimizing both specificity and affinity. Nucleic Acids Res..

[17] Li X., Chung L.W., Li G. (2016). Multiscale simulations on spectral tuning and the photoisomerization mechanism in fluorescent RNA spinach. J. Chem. Theory Comput..

[18] Kumar P., Kumar A. (2020). Nucleobase sequence based building up of reliable QSAR models with the index of ideality correlation using Monte Carlo method. J. Biomol. Struct. Dyn..

[19] Boushaba K., Levine H., Hamilton M.N. (2009). A mathematical feasibility argument for the use of aptamers in chemotherapy and imaging. Math. Biosci..

[20] Chen X., Ellington A.D. (2009). Design principles for ligand-sensing, conformation-switching ribozymes. PLoS Comput. Biol..

[21] Avihoo A., Gabdank I., Shapira M., Barash D. (2007). In silico design of small RNA switches. IEEE Trans. Nanobiosci..

[22] Domin G., Findeiß S., Wachsmuth M., Will S., Stadler P.F., Mörl M. (2017). Applicability of a computational design approach for synthetic riboswitches. Nucleic Acids Res..

[23] Findeiß S., Etzel M., Will S., Mörl M., Stadler P.F. (2017). Design of artificial riboswitches as biosensors. Sensors.

[24] Gong S., Wang Y., Wang Z., Zhang W. (2017). Computational methods for modeling aptamers and designing riboswitches. Int. J. Mol. Sci..

[25] Boussebayle A., Torka D., Ollivaud S., Braun J., Bofill-Bosch C., Dombrowski M., Groher F., Hamacher K., Suess B. (2019). Next-level riboswitch development-implementation of Capture-SELEX facilitates identification of a new synthetic riboswitch. Nucleic Acids Res..

[26] Tseng C.Y., Ashrafuzzaman M., Mane J.Y., Kapty J., Mercer J.R., Tuszynski J.A. (2011). Entropic fragment-based approach to aptamer design. Chem. Biol. Drug Des..

[27] Zavyalova E., Golovin A., Reshetnikov R., Mudrik N., Panteleyev D., Pavlova G., Kopylov A. (2011). Novel modular DNA aptamer for human thrombin with high anticoagulant activity. Curr. Med. Chem..

[28] Varizhuk A.M., Tsvetkov V.B., Tatarinova O.N., Kaluzhny D.N., Florentiev V.L., Timofeev E.N., Shchyolkina A.K., Borisova O.F., Smirnov I.P., Grokhovsky S.L. (2013). Synthesis, characterization and in vitro activity of thrombin-binding DNA aptamers with triazole internucleotide linkages. Eur. J. Med. Chem..

[29] Tatarinova O., Tsvetkov V., Basmanov D., Barinov N., Smirnov I., Timofeev E., Kaluzhny D., Chuvilin A., Klinov D., Varizhuk A. (2014). Comparison of the ‘chemical’ and ‘structural’ approaches to the optimization of the thrombin-binding aptamer. PLoS ONE.

[30] Mahmood M.A., Ali W., Adnan A., Iqbal S.M. (2014). 3D structural integrity and interactions of single-stranded protein-binding DNA in a functionalized nanopore. J. Phys. Chem. B.

[31] Rangnekar A., Nash J.A., Goodfred B., Yingling Y.G., LaBean T.H. (2016). Design of potent and controllable anticoagulants using DNA aptamers and nanostructures. Molecules.

[32] Van Riesen A.J., Fadock K.L., Deore P.S., Desoky A., Manderville R.A., Sowlati-Hashjin S., Wetmore S.D. (2018). Manipulation of a DNA aptamer-protein binding site through arylation of internal guanine residues. Org. Biomol. Chem..

[33] Sgobba M., Olubiyi O., Ke S., Haider S. (2012). Molecular dynamics of HIV1-integrase in complex with 93del—A structural perspective on the mechanism of inhibition. J. Biomol. Struct. Dyn..

[34] Do N.Q., Lim K.W., Teo M.H., Heddi B., Phan A.T. (2011). Stacking of G-quadruplexes: NMR structure of a G-rich oligonucleotide with potential anti-HIV and anticancer activity. Nucleic Acids Res..

[35] Aeksiri N., Songtawee N., Gleeson M.P., Hannongbua S., Choowongkomon K. (2014). Insight into HIV-1 reverse transcriptase-aptamer interaction from molecular dynamics simulations. J. Mol. Model..

[36] Nguyen P.D.M., Zheng J., Gremminger T.J., Qiu L., Zhang D., Tuske S., Lange M.J., Griffin P.R., Arnold E., Chen S.J. (2020). Binding interface and impact on protease cleavage for an RNA aptamer to HIV-1 reverse transcriptase. Nucleic Acids Res..

[37] Song Y., Song J., Wei X., Huang M., Sun M., Zhu L., Lin B., Shen H., Zhu Z., Yang C. (2020). Discovery of aptamers targeting the receptor-binding domain of the SARS-CoV-2 spike glycoprotein. Anal. Chem..

[38] Sabri M.Z., Abdul Hamid A.A., Sayed Hitam S.M., Abdul Rahim M.Z. (2019). In silico screening of aptamers configuration against hepatitis B surface antigen. Adv. Bioinform..

[39] Soon S., Nordin N.A. (2019). In silico predictions and optimization of aptamers against Streptococcus agalactiae surface protein using computational docking. Mater. Today Proc..

[40] Rockey W.M., Hernandez F.J., Huang S.Y., Cao S., Howell C.A., Thomas G.S., Liu X.Y., Lapteva N., Spencer D.M., McNamara J.O. (2011). Rational truncation of an RNA aptamer to prostate-specific membrane antigen using computational structural modeling. Nucleic Acid Ther..

[41] Bavi R., Liu Z., Han Z., Zhang H., Gu Y. (2019). In silico designed RNA aptamer against epithelial cell adhesion molecule for cancer cell imaging. Biochem. Biophys. Res. Commun..

[42] Bell D.R., Weber J.K., Yin W., Huynh T., Duan W., Zhou R. (2020). In silico design and validation of high-affinity RNA aptamers targeting epithelial cellular adhesion molecule dimers. Proc. Natl. Acad. Sci. USA.

[43] Wang Q.L., Cui H.F., Du J.F., Lv Q.Y., Songa X. (2019). In silico post-SELEX screening and experimental characterizations for acquisition of high affinity DNA aptamers against carcinoembryonic antigen. RSC Adv..

[44] Santini B.L., Zúñiga-Bustos M., Vidal-Limon A., Alderete J.B., Águila S.A., Jiménez V.A. (2020). In silico design of novel mutant anti-MUC1 aptamers for targeted cancer therapy. J. Chem. Inf. Model..

[45] Knight C.G., Platt M., Rowe W., Wedge D.C., Khan F., Day P.J., McShea A., Knowles J., Kell D.B. (2009). Array-based evolution of DNA aptamers allows modelling of an explicit sequence-fitness landscape. Nucleic Acids Res..

[46] Hu W.P., Kumar J.V., Huang C.J., Chen W.Y. (2015). Computational selection of RNA aptamer against angiopoietin-2 and experimental evaluation. Biomed. Res. Int..

[47] Cataldo R., Ciriaco F., Alfinito E. (2018). A validation strategy for in silico generated aptamers. Comput. Biol. Chem..

[48] Shcherbinin D.S., Gnedenko O.V., Khmeleva S.A., Usanov S.A., Gilep A.A., Yantsevich A.V., Shkel T.V., Yushkevich I.V., Radko S.P., Ivanov A.S. (2015). Computer-aided design of aptamers for cytochrome p450. J. Struct. Biol..

[49] Ahirwar R., Nahar S., Aggarwal S., Ramachandran S., Maiti S., Nahar P. (2016). In silico selection of an aptamer to estrogen receptor alpha using computational docking employing estrogen response elements as aptamer-alike molecules. Sci. Rep..

[50] Heiat M., Najafi A., Ranjbar R., Latifi A.M., Rasaee M.J. (2016). Computational approach to analyze isolated ssDNA aptamers against angiotensin II. J. Biotechnol..

[51] Rabal O., Pastor F., Villanueva H., Soldevilla M.M., Hervas-Stubbs S., Oyarzabal J. (2016). In silico aptamer docking studies: From a retrospective validation to a prospective case study-TIM3 aptamers binding. Mol. Ther. Nucleic Acids..

[52] Genheden S., Ryde U. (2015). The MM/PBSA and MM/GBSA methods to estimate ligand-binding affinities. Expert Opin. Drug Discov..

[53] Lietard J., Abou Assi H., Gómez-Pinto I., González C., Somoza M.M., Damha M.J. (2017). Mapping the affinity landscape of Thrombin-binding aptamers on 2′F-ANA/DNA chimeric G-Quadruplex microarrays. Nucleic Acids Res..

[54] Lu X., Olson W.K. (2003). 3DNA: A software package for the analysis, rebuilding, and visualization of three-dimensional nucleic acid structures. Nucleic Acids Res..

[55] Pronk S., Pall S., Schulz R., Larsson P., Bjelkmar P., Apostolov R., Shirts M.R., Smith J.C., Kasson P.M., van der Spoel D. (2013). GROMACS 4.5: A high-throughput and highly parallel open source molecular simulation toolkit. Bioinformatics.

[56] Sorin E.J., Pande V.S. (2005). Exploring the helix-coil transition via all-atom equilibrium ensemble simulations. Biophys. J..

[57] Pérez A., Marchán I., Svozil D., Sponer J., Cheatham T.E., Laughton C.A., Orozco M. (2007). Refinement of the AMBER force field for nucleic acids: Improving the description of alpha/gamma conformers. Biophys. J..

[58] Hornak V., Abel R., Okur A., Strockbine B., Roitberg A., Simmerling C. (2006). Comparison of multiple amber force fields and development of improved protein backbone parameters. Proteins.

[59] Tsvetkov V., Varizhuk A., Pozmogova G., Smirnov I., Kolganova N., Timofeev E. (2015). A universal base in a specific role: Tuning up a thrombin aptamer with 5-nitroindole. Sci. Rep..

[60] Phillips J.C., Braun R., Wang W., Gumbart J., Tajkhorshid E., Villa E., Chipot C., Skeel R.D., Kale L., Schulten K. (2005). Scalable molecular dynamics with NAMD. J. Comput. Chem..

[61] Brooks B.R., Brooks C.L., Mackerell A.D., Nilsson L., Petrella R.J., Roux B., Won Y., Archontis G., Bartels C., Boresch S. (2009). CHARMM: The biomolecular simulation program. J. Comput. Chem..

[62] Maier J.A., Martinez C., Kasavajhala K., Wickstrom L., Hauser K.E., Simmerling C. (2015). ff14SB: Improving the accuracy of protein side chain and backbone parameters from ff99SB. J. Chem. Theory Comput..

[63] Ritchie D.W., Venkatraman V. (2010). Ultra-fast FFT protein docking on graphics processors. Bioinformatics.

[64] Kumari R., Kumar R., Lynn A., Open Source Drug Discovery Consortium (2014). g_mmpbsa—A GROMACS tool for high-throughput MM-PBSA calculations. J. Chem. Inf. Model..

[65] Xu X., Zhao P., Chen S.J. (2014). Vfold: A web server for RNA structure and folding thermodynamics prediction. PLoS ONE.

[66] Zhang D., Chen S.J. (2018). IsRNA: An iterative simulated reference state approach to modeling correlated interactions in RNA folding. J. Chem. Theor. Comput..

[67] Xu X., Qiu L., Yan C., Ma Z., Grinter S.Z., Zou X. (2017). Performance of MDockPP in CAPRI rounds 28–29 and 31–35 including the prediction of water-mediated interactions. Proteins.

[68] Benfenati E., Toropov A.A., Toropova A.P., Manganaro A., Gonella Diaza R. (2011). coral software: QSAR for anticancer agents. Chem. Biol. Drug Des..

[69] Musafia B., Oren-Banaroya R., Noiman S. (2014). Designing anti-influenza aptamers: Novel quantitative structure activity relationship approach gives insights into aptamer-virus interaction. PLoS ONE.

[70] Song J., Zheng Y., Huang M., Wu L., Wang W., Zhu Z., Song Y., Yang C. (2020). A Sequential Multidimensional Analysis Algorithm for Aptamer Identification based on Structure Analysis and Machine Learning. Anal. Chem..

[71] Vanommeslaeghe K., Hatcher E., Acharya C., Kundu S., Zhong S., Shim J., Darian E., Guvench O., Lopes P., Vorobyov I. (2010). CHARMM general force field: A force field for drug-like molecules compatible with the CHARMM all-atom additive biological force fields. J. Comput. Chem..

[72] Wang J., Wang J., Huang Y., Xiao Y. (2019). 3dRNA v2.0: An updated Web server for RNA 3D structure prediction. Int. J. Mol. Sci..

[73] Bellaousov S., Reuter J.S., Seetin M.G., Mathews D.H. (2013). RNAstructure: Web servers for RNA secondary structure prediction and analysis. Nucleic Acids Res..

[74] Case D.A., Cheatham T.E., Darden T., Gohlke H., Luo R., Merz K.M., Onufriev A., Simmerling C., Wang B., Woods R.J. (2005). The Amber biomolecular simulation programs. J. Comput. Chem..

[75] Huang S.Y., Zou X. (2010). MDockPP: A hierarchical approach for protein-protein docking and its application to CAPRI rounds 15-19. Proteins.

[76] Patriarca C., Macchi R.M., Marschner A.K., Mellstedt H. (2012). Epithelial cell adhesion molecule expression (CD326) in cancer: A short review. Cancer Treat. Rev..

[77] Lorenz R., Bernhart S.H., Höner Zu Siederdissen C., Tafer H., Flamm C., Stadler P.F., Hofacker I.L. (2011). ViennaRNA package 2.0. Algorithms Mol. Biol..

[78] Aliev A.E., Kulke M., Khaneja H.S., Chudasama V., Sheppard T.D., Lanigan R.M. (2014). Motional timescale predictions by molecular dynamics simulations: Case study using proline and hydroxyproline sidechain dynamics. Proteins.

[79] Bavi R., Kumar R., Choi L., Woo Lee K. (2016). Exploration of novel inhibitors for bruton’s tyrosine kinase by 3D QSAR modeling and molecular dynamics simulation. PLoS ONE.

[80] Cheatham T.E., Case D.A. (2013). Twenty-five years of nucleic acid simulations. Biopolymers.

[81] Roberts V.A., Thompson E.E., Pique M.E., Perez M.S., Ten Eyck L.F. (2013). DOT2: Macromolecular docking with improved biophysical models. J. Comput. Chem..

[82] Pierce B.G., Wiehe K., Hwang H., Kim B.H., Vreven T., Weng Z. (2014). ZDOCK server: Interactive docking prediction of protein-protein complexes and symmetric multimers. Bioinformatics.

[83] Markham N.R., Zuker M. (2008). UNAFold: Software for nucleic acid folding and hybridization. Methods Mol. Biol..

[84] Sato K., Hamada M., Asai K., Mituyama T. (2009). CentroidFold: A web server for RNA secondary structure prediction. Nucleic Acids Res..

[85] Popenda M., Szachniuk M., Antczak M., Purzycka K.J., Lukasiak P., Bartol N., Blazewicz J., Adamiak R.W. (2012). Automated 3D structure composition for large RNAs. Nucleic Acids Res..

[86] Boniecki M.J., Lach G., Dawson W.K., Tomala K., Lukasz P., Soltysinski T., Rother K.M., Bujnicki J.M. (2016). SimRNA: A coarse-grained method for RNA folding simulations and 3D structure prediction. Nucleic Acids Res..

[87] Trott O., Olson A.J. (2010). AutoDock Vina: Improving the speed and accuracy of docking with a new scoring function: Efficient optimization and multithreading. J. Comput. Chem..

[88] Allen W.J., Balius T.E., Mukherjee S., Brozell S.R., Moustakas D.T., Lang P.T., Case D.A., Kuntz I.D., Rizzo R.C. (2015). DOCK 6: Impact of new features and current docking performance. J. Comput. Chem..

[89] Vries S.J., de Dijk M., van Bonvin A.M. (2010). The HADDOCK web server for data-driven biomolecular docking. Nat. Protoc..

[90] Schneidman-Duhovny D., Inbar Y., Nussinov R., Wolfson H.J. (2005). PatchDock and SymmDock: Servers for rigid and symmetric docking. Nucleic Acids Res..

[91] Zuker M. (2003). Mfold web server for nucleic acid folding and hybridization prediction. Nucleic Acids Res..

[92] Cheng C.Y., Chou F.C., Das R. (2015). Modeling complex RNA tertiary folds with Rosetta. Methods Enzymol..

[93] Huang Y., Liu S., Guo D., Li L., Xiao Y. (2013). A novel protocol for three-dimensional structure prediction of RNA-protein complexes. Sci. Rep..

[94] Bauer M., Strom M., Hammond D.S., Shigdar S. (2019). Anything you can do, I can do better: Can aptamers replace antibodies in clinical diagnostic applications?. Molecules.

[95] Ilgu M., Yan S., Khounlo R.M., Lamm M.H., Nilsen-Hamilton M. (2019). Common secondary and tertiary structural features of aptamer-ligand interaction shared by RNA aptamers with different primary sequences. Molecules.

[96] Khoshbin Z., Housaindokht M.R. (2020). Computer-aided aptamer design for sulfadimethoxine antibiotic: Step by step mutation based on MD simulation approach. J. Biomol. Struct. Dyn..

[97] Gruber A.R., Lorenz R., Bernhart S.H., Neubock R., Hofacker I.L. (2008). The Vienna RNA Websuite. Nucleic Acids Res..

[98] Bhagwat M., Aravind L. (2007). PSI-BLAST tutorial. Methods Mol. Biol..

[99] Housaindokht M.R., Bozorgmehr M.R., Bahrololoom M. (2008). Analysis of ligand binding to proteins using molecular dynamics simulations. J. Theor. Biol..

[100] Kim N., Gan H.H., Schlick T. (2007). A computational proposal for designing structured RNA pools for in vitro selection of RNAs. RNA.

[101] Luo X., McKeague M., Pitre S., Dumontier M., Green J., Golshani A., Derosa M.C., Dehne F. (2010). Computational approaches toward the design of pools for the in vitro selection of complex aptamers. RNA.

[102] Ashrafuzzaman M., Tseng C.Y., Kapty J., Mercer J.R., Tuszynski J.A. (2013). A computationally designed DNA aptamer template with specific binding to phosphatidylserine. Nucleic Acid Ther..

[103] Jokar M., Safaralizadeh M.H., Hadizadeh F., Rahmani F., Kalani M.R. (2017). Apta-nanosensor preparation and in vitro assay for rapid diazinon detection using a computational molecular approach. J. Biomol. Struct. Dyn..

[104] Ruan M., Seydou M., Noel V., Piro B., Maurel F., Barbault F. (2017). Molecular dynamics simulation of a RNA aptasensor. J. Phys. Chem. B.

[105] Belinskaia D.A., Avdonin P.V., Avdonin P.P., Jenkins R.O., Goncharov N.V. (2019). Rational in silico design of aptamers for organophosphates based on the example of paraoxon. Comput. Biol. Chem..

[106] Carothers J.M., Oestreich S.C., Davis J.H., Szostak J.W. (2004). Informational complexity and functional activity of RNA structures. J. Am. Chem. Soc..

[107] Duan Y., Wu C., Chowdhury S., Lee M.C., Xiong G., Zhang W.E.I., Yang R., Cieplak P., Luo R.A.Y., Lee T. (2003). A point-charge force field for molecular mechanics simulations of proteins based on condensed-phase quantum mechanical calculations. J. Comput. Chem..

[108] Kikin O., D’Antonio L., Bagga P.S. (2006). QGRS Mapper: A web-based server for predicting G-quadruplexes in nucleotide sequences. Nucleic Acids Res..

[109] Lindorff-Larsen K., Piana S., Palmo K., Maragakis P., Klepeis J., Dror R.O., Shaw D.E. (2010). Improved side-chain torsion potentials for the amber ff99SB protein force field. Proteins.

[110] Wang J., Wolf R.M., Caldwell J.W., Kollman P.A., Case D.A. (2004). Development and testing of a general amber force field. J. Comput. Chem..

[111] Morris G.M., Goodsell D.S., Halliday R.S., Huey R., Hart W.E., Belew R.K., Olson A.J. (1998). Automated docking using a Lamarckian genetic algorithm and an empirical binding free energy function. J. Comput. Chem..

[112] Lin P.H., Tsai C.W., Wu J.W., Ruaan R.C., Chen W.Y. (2012). Molecular dynamics simulation of the induced-fit binding process of DNA aptamer and L-argininamide. Biotechnol. J..

[113] Albada H.B., Golub E., Willner I. (2015). Computational docking simulations of a DNA-aptamer for argininamide and related ligands. J. Comput. Aided Mol. Des..

[114] Verdonck L., Buyst D., de Vries A.M., Gheerardijn V., Madder A., Martins J.C. (2018). Tethered imidazole mediated duplex stabilization and its potential for aptamer stabilization. Nucleic Acids Res..

[115] Wachsmuth M., Findeiß S., Weissheimer N., Stadler P.F., Mörl M. (2013). De novo design of a synthetic riboswitch that regulates transcription termination. Nucleic Acids Res..

[116] Zhou Q., Xia X., Luo Z., Liang H., Shakhnovich E. (2015). Searching the sequence space for potent aptamers using SELEX in silico. J. Chem. Theory Comput..

[117] Jokar M., Safaralizadeh M.H., Hadizadeh F., Rahmani F., Kalani M.R. (2016). Design and evaluation of an apta-nano-sensor to detect acetamiprid in vitro and in silico. J. Biomol. Struct. Dyn..

[118] Tomita Y., Morita Y., Suga H., Fujiwara D. (2016). DNA module platform for developing colorimetric aptamer sensors. Biotechniques.

[119] Hilder T.A., Hodgkiss J.M. (2017). The bound structures of 17β-estradiol-binding aptamers. Chemphyschem.

[120] Zhao M., Li W., Liu K., Li H., Lan X. (2019). C4-HSL aptamers for blocking qurom sensing and inhibiting biofilm formation in *Pseudomonas aeruginosa* and its structure prediction and analysis. PLoS ONE.

